# Displacement of Small bowel into the Lesser Sac in a Case of Complete Congenital Pouch Colon

**Published:** 2015-01-10

**Authors:** Bilal Mirza, Muhammad Saleem

**Affiliations:** Department of Pediatric Surgery, The Children’s Hospital and the Institute of Child Health, Lahore, Pakistan

**Keywords:** Congenital pouch colon, Internal hernia, Lesser sac

## Abstract

Postoperative neonatal intestinal obstruction has a myriad of etiology. An operated case of imperforate anus developed intestinal obstruction early postoperatively. At re-operation, missed complete congenital pouch colon with a small perforation and displacement of small bowel into the lesser sac were found. The patient was managed by adhesionolysis, excision of pouch colon, and end ileostomy. The patient did well postoperatively.

## CASE REPORT

An 8-day-old male neonate, known case of imperforate anus with a colostomy at the left lower quadrant, was referred to our hospital with abdominal distension, bilious vomiting, and not passing stool for 3 days. History revealed that the colostomy was formed on 2nd day of life at another hospital which started working on the following day. The patient however developed abdominal distension and bilious vomiting on 3rd postoperative day followed by failure to pass stool from the stoma. After failure of conservative measures, the patient was referred to us. 

Abdominal radiograph showed large gas shadows and air fluid levels. Ultrasound of the abdomen depicted dilated bowel loops with absent peristalsis. Laboratory tests showed Hemoglobin of 10g/dl and platelets count of 65000/mm3. Other laboratory parameters were normal. 

After resuscitation and optimization, the patient was operated, which showed fecal peritonitis and two cystic structures in the abdominal cavity. On dissection of upper cystic structure, few loops of small bowel were found trapped beneath the stomach (lesser sac) and appeared herniated through a well appreciable opening (Fig.1). Further dissection showed that opening was formed by free margin of greater omentum which was adhered with bowel loops and posterior abdominal wall. There was no actual defect in the greater omentum. In the lesser sac, the small bowel loops were adherent with each other and the posterior wall of stomach; feculent colored exudate was visible over bowel loops (Fig.2). The displaced and adherent bowel loops were viable; adhesionolysis was performed. The second cystic structure was a complete pouch colon with a small perforation (Fig.3). A wall of pouch colon was exteriorized as window colostomy during first surgery in peripheral centre. The pouch colon was mobilized and excised with repair of colo-vesical fistula; end ileostomy was done. The postoperative course remained uneventful. The patient was discharged on 7th postoperative day. The patient is in our follow-up for further procedures.

**Figure:**
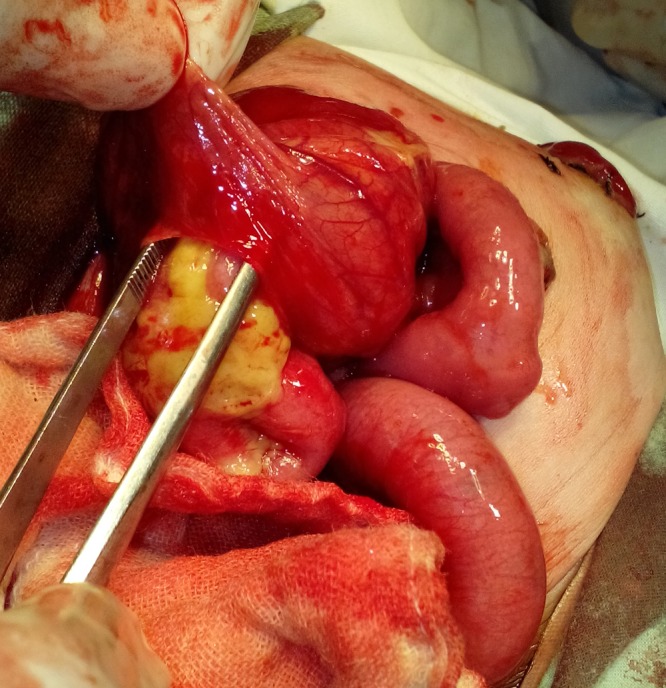
Small bowel loops adherent and jumbled up below the stomach and greater omentum adherent simulating as a true opening.

**Figure:**
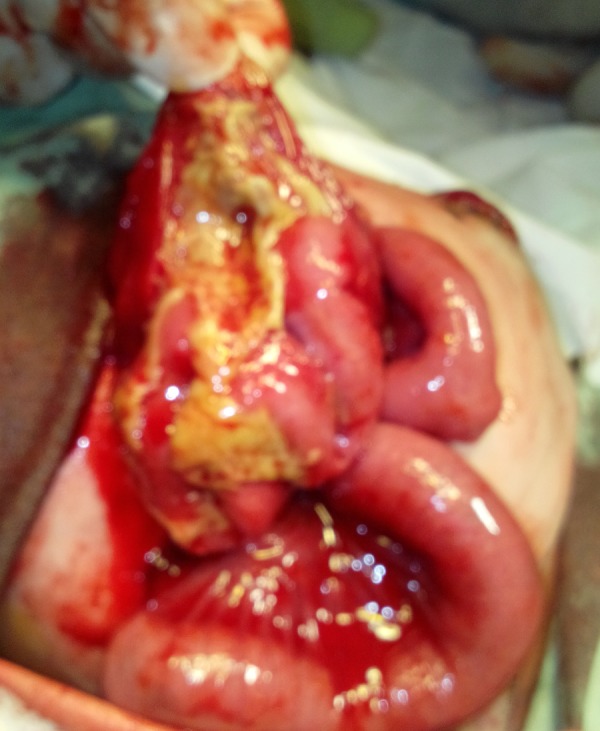
Figure 2: Gut loops dissected out of lesser sac.

**Figure:**
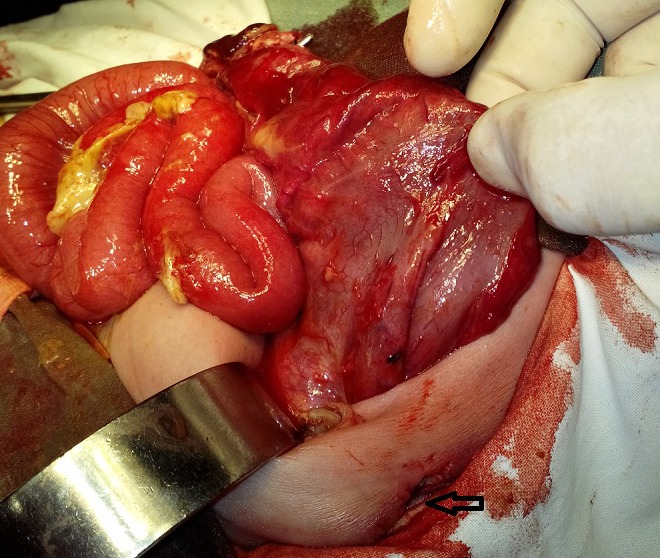
Figure 3: Complete congenital pouch colon a wall of which was exteriorized as window colostomy (Arrow).

## DISCUSSION

Earlier we have studied etiology of first week postoperative intestinal obstruction in neonates and children and found a host of etiologies including postoperative intussusception, internal herniation through a mesenteric defect, soft adhesions causing intestinal kinking, gossypiboma, and missed incomplete congenital pouch colon (CPC).[1] In the index case, missed complete CPC with a small perforation and displacement of small bowel into the lesser sac were the etiologies of first week postoperative intestinal obstruction.

Internal herniation may be paraduodenal, transmesenteric or transmesocolic, paracolic, and transomental etc. Herniation to lesser sac may occur through natural opening i.e. foramen of Winslow, transmesocolic, transgastrocolic ligament, through greater omentum, and after Roux-en-Y gastric bypass.[2-5] In our case, translocation of small bowel into the lesser sac was not through the above cited routes; in fact, free margin of greater omentum was adherent to the gut loops and posterior abdominal wall thus simulated an opening. 

While probing the etiology of entrapment of small bowel loops into the lesser sac in the index case, following points should be considered; the absent or malformed gastrocolic ligament owing to complete CPC would have allowed free movement of small bowel behind stomach; and perforation peritonitis might have resulted in adhesions between greater omentum small bowel loops, and stomach thus entrapping the small bowel there. 

The presentation of internal herniation may range from pain abdomen to as sinister as intestinal gangrene and perforation peritonitis.[2-5] The index case presented with signs of intestinal obstruction within the first week of operation. Moreover, the entrapment did not lead to perforation; the missed complete CPC might had not emptied properly through window stoma and resulted in its small perforation or a delay in forming stoma in the index case (2nd day of life) might be the reason. Nevertheless, X-ray did not show pneumoperitoneum. Retention of CPC during initial surgery is associated with a number of complications which often proved fatal.[6] In our case, the operative notes of first surgery were silent as to CPC which may indicate that it was missed and not retained intentionally. The ideal staged treatment of CPC is excision of the pouch with end ileostomy, [6] which we later did during exploration at our department.

## Footnotes

**Source of Support:** Nil

**Conflict of Interest:** None

